# Global Cue Inconsistency Diminishes Learning of Cue Validity

**DOI:** 10.3389/fpsyg.2016.01743

**Published:** 2016-11-11

**Authors:** Tony S. L. Wang, Nicole Christie, Piers D. L. Howe, Daniel R. Little

**Affiliations:** ^1^Cognitive, Linguistics and Psychological Sciences, Brown University, ProvidenceRI, USA; ^2^School of Psychological Sciences, The University of Melbourne, MelbourneVIC, Australia

**Keywords:** learning, probabilistic feedback, search behavior, selective attention, exploration, exploitation

## Abstract

In daily life, we make decisions that are associated with probabilistic outcomes (e.g., the chance of rain today). People search for and utilize information that validly predicts an outcome (i.e., we look for dark clouds to indicate the possibility of rain). In the current study (*N* = 107), we present a two-stage learning task that examines how participants learn and utilize predictive information within a probabilistic learning environment. In the first stage, participants select one of three cues that gives predictive information about the outcome of the second stage. Participants then use this information to predict the outcome in stage two, for which they receive feedback. Critically, only one of the three cues in stage one gives valid predictive information about the outcome in stage two. Participants must differentiate the valid from non-valid cues and select this cue on subsequent trials in order to inform their prediction of the outcome in stage two. The validity of this predictive information, however, is reinforced with varying levels of probabilistic feedback (i.e., 75, 85, 95, 100%). A second manipulation involved changing the consistency of the predictive information in stage one and the outcome in stage two. The results show that participants, with higher levels of probabilistic feedback, learned to utilize the valid cue. In inconsistent task conditions, however, participants were significantly less successful in utilizing higher validity cues. We interpret this result as implying that learning in probabilistic categorization is based on developing a representation of the task that allows for goal-directed action.

## Introduction

When searching for resources (e.g., time, food, money, information), people are often confronted with an exploitation-exploration dilemma. For example, a businessman in a foreign city might eat at the same restaurant each evening because the food is consistently good. On the other hand, if the first restaurant varied the quality of their food from evening to evening, the diner might be tempted to visit a different restaurant. This exploitation-exploration dilemma illustrates the balance between searching for resources and then utilizing those resources once found (Cohen et al., 2007). In addition to other factors (e.g., motivation; Markman et al., 2005; Maddox et al., 2006), a variety of research fields in cognitive psychology investigating search behavior, such as associative learning, categorisation, and decision-making, have identified uncertainty (Daw et al., 2005, 2006) as a key factor that influence the adoption of an exploitation or exploration strategy.

Exploitation can be thought of as the process of using previously obtained information to acquire known rewards (Dam and Körding, 2009). Decision makers are adept at utilizing information that is highly predictive of a positive outcome in order to maximize reward (Bröder, 2000; Rieskamp and Otto, 2006). In one study, participants were asked to predict the more creditworthy company from two unnamed companies (Rieskamp and Otto, 2006) based on a list of company attributes (e.g., efficiency, financial resources, financial flexibility). The probability with which each attribute correctly predict superior creditworthiness was also shown to participants. Rieskamp and Otto (2006) showed that decisions were based on attributes with the highest validity that discriminated the two companies (e.g., company A is superior to company B on efficiency). This indicates that participants utilize cues that are highly predictive of the desired outcome at the expense of other cues.

How decision makers learn to utilize attributes has been studied in a variety of related domains such as category and multiple cue probability learning with a focus on the role of selective attention (for a review see Kruschke and Johansen, 1999). In general, predictive attributes are utilized most often, and attention to predictive attributes increases over time with experience. Within a probabilistic learning environment, however, predictive attributes do not necessarily correctly predict the desired outcome on all trials. This reduces the utilization of predictive attributes and promotes the utilization of non-predictive or irrelevant attributes. As a consequence, attention to predictive attributes may be reduced within a probabilistic environment.

Little and Lewandowsky (2009) showed that learning a probabilistic task resulted in a broadening of selective attention that improved recall of the attended attributes. In their Experiment 2, participants classified stimuli varying along four perceptual features (X, Y, Z, and C). Two attributes (X and Y) determined the correct classification via an XOR rule. The non-diagnostic features Z and C were correlated with each other across all trials. Participants utilized the XY pair more when feedback was deterministic (i.e., 100% validity) than probabilistic (i.e., 75% validity). To examine selective attention, participants completed a feature completion task following initial category learning in which missing properties of the stimulus were predicted from a category label and a partial stimulus. Little and Lewandowsky (2009) found better prediction in the probabilistic than in the deterministic group, who displayed only near chance feature completion accuracy. This supports the idea that probabilistic feedback shifts attention away from diagnostic features (i.e., increasing exploration) resulting in better memory for non-diagnostic features in the probabilistic than in the deterministic condition.

In repeated choice tasks with probabilistic feedback in which information about the underlying probabilities needs to be acquired on a trial-by-trial basis, participants often switch to an exploration strategy. For example, given a simple choice between 70% chance of winning $1 and 30% chance of winning $1, it is obvious to take the higher probability option. People, however, might take the lower probability option if the choice is repeatedly presented over a sequence of decisions and if the probabilities are learned via experience rather than explicitly stated (e.g., Gaissmaier and Schooler, 2008; James and Koehler, 2011). This behavior is called probability matching as the probability with which an option is selected matches actual probability of the outcome being correct. Probability matching is sub-optimal since the long-term payoff of selecting the better option probabilistically is lower than if only selecting the better option (i.e., maximizing). The shift from maximizing to probability matching is consistent with exploratory behavior since reward is sacrificed in order to find potentially superior options. Interestingly, participants are more likely to adopt probability matching if they believe there is an underlying pattern or causal structure within the probabilistic environment (Gaissmaier and Schooler, 2008; James and Koehler, 2011). Thus, whether an exploitation or exploration strategy is adopted depends on the task structure and learning environment.

In the current paper, we attempted to extend the conclusion offered by Little and Lewandowsky (2009) and determine the effect of parametrically varying probabilistic feedback on utilization (i.e., the probability that an option is correct when selected varied from 0.75, 0.85, 0.95, and 1.0; Little and Lewandowsky (2009) only used the highest and lowest values). We further manipulated the task structure by varying the causal structure of the task. We seek to understand how the interaction of probabilistic feedback and causal structure affect how easily people learn to utilize and exploit valid cues.

The task described by Little and Lewandowsky (2009) does not allow an online measurement of attention and attribute utilization, rather the authors inferred greater attention to irrelevant features from superior feature completion accuracy in the probabilistic condition. Previous studies (i.e., Kruschke et al., 2005; Rehder and Hoffman, 2005) have used eyetracking to directly index attention during category learning tasks, and showed that participants increase their attention to predictive attributes. Specifically, eyegaze fixation has been shown to follow probabilistic feedback (McColeman et al., 2011) with more fixations to irrelevant cues in lower probabilistic feedback conditions.

**FIGURE 1:**
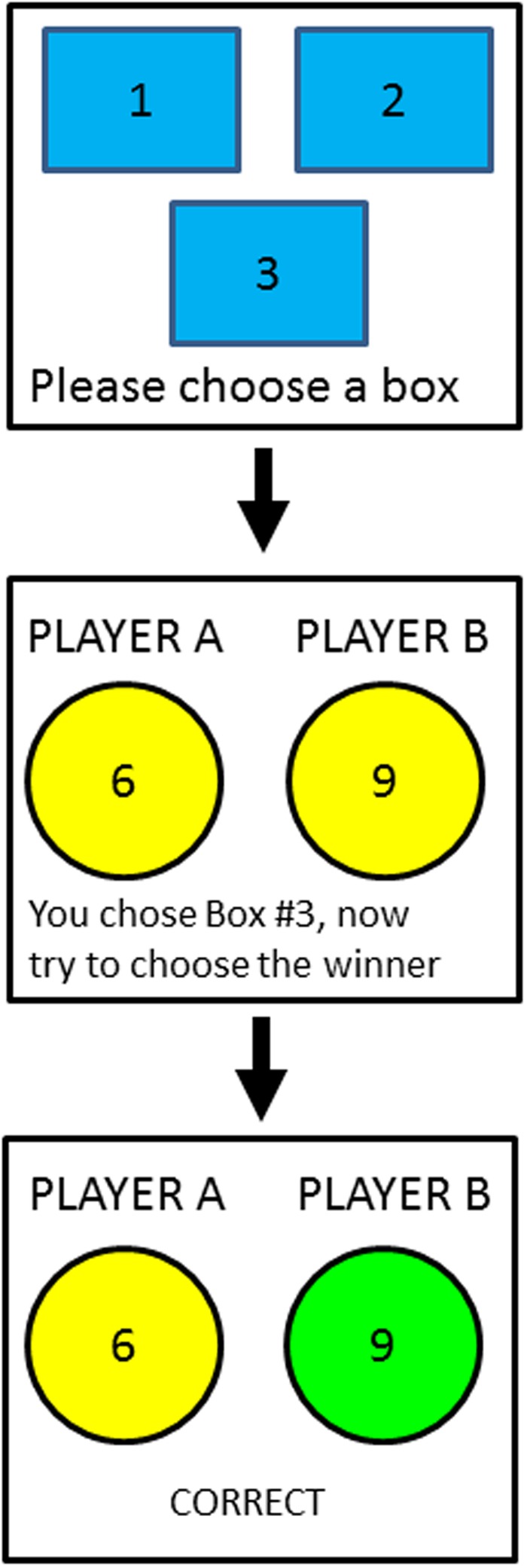
**Schematic diagram of the task. (Top)** At the beginning of a trial, the participant must choose a box to examine from the three available boxes. One of the boxes, chosen randomly for each participant, has validity greater than 0.5; the remaining two boxes have validities equal to 0.5. **(Middle)** After choosing a box, the participant is shown the token allocation of each player and must choose the winning player (i.e., the player who had more tokens in at least two of the three boxes). In the consistent condition, the winning player is the player with more tokens in the valid box; in the inconsistent condition, the winning player is the player with fewer tokens in the valid box. **(Bottom)** After choosing a player, the participant is shown feedback indicating whether her response was correct or incorrect. The winning player was determined randomly according to the validity of the valid box.

In our study, we designed a two-stage learning game to index attribute utilization and attention in a probabilistic learning environment. We created a variant of the Colonel Blotto game (Gross and Wagner, 1950; Borel, 1953) in which there are two artificial (computer-generated) players, each with a variable number of tokens. Each player allocates tokens to three boxes, and the overall winner is the player who wins two or more boxes (usually the player with the most tokens in the box). The participants in our experiment act as the ‘referee’ by selecting one box to examine, and judging, based on the outcome of that box alone, which of the two players they think won more boxes overall (**Figure 1**). In the context of our initial example, the participant is the diner and each of the “players” represents a restaurant that varies on three attributes. The critical aspect of our task is that two boxes represent invalid cues in that the winner of either box is not correlated with the overall winner (i.e., 0.5 validity). Conversely, the third box has a positive validity that varies across four conditions from 0.75, 0.85, 0.95 to 1.0. Note, participants do not know the total number of tokens given to each player on each trial and how each player allocated their tokens to each box. Thus, it is impossible for participants to deduce the winner of the other two unselected boxes. The validity of each box is held constant, and in order to *solve* the task, participants must learn that the winner of one of the three boxes is more likely to win the round.

We chose to use this task in order to explicitly monitor which information source was utilized on each trial rather than inferring attentional distributions via model parameters (e.g., Little and Lewandowsky, 2009). Here we can simply count the number of times each box was chosen. We expect to find a positive correlation between valid box validity and valid box utilization (Kruschke and Johansen, 1999; Little and Lewandowsky, 2009). In fact, the optimal behavior is to exploit the valid box. However, as the valid box validity decreases, participants might engage in probability matching and select the valid box less often even though it is not optimal to do so.

Several researchers (Shanks, 2007 and Mitchell et al., 2009 for review) have claimed that the learning process in the current categorization task can be accounted for by simple associative learning mechanisms (i.e., Rescorla and Wagner, 1972). Box utilization can be modeled using a simple reinforcement learning model (i.e., Sutton and Barto, 1998; Dayan and Niv, 2008), with an attention weight parameter to each box being adjusted based on the strength of the association between the winner of the box and the overall winner (i.e., Mackintosh, 1975). Specifically, an associative link between the winner of the selected box and round winner is formed in memory. Contrary to the reinforcement learning model, participants may develop a causal belief in which they expect the winner of the selected box to win at least one of the remaining two boxes. Under the causal model, reversing the relationship between box winner and overall winner impairs performance since it contradicts participants’ causal belief. According to the reinforcement learning model, however, such a manipulation will not impair performance.

In the current experiment, we introduced a second factor in which either the *winner* or *loser* of the valid box was the overall winner. In the *consistent* condition, a participant should choose the player with the *highest* number of tokens in the valid box. In the *inconsistent* condition, the only difference was that the overall winner was determined by whoever had the *fewest* number of tokens in the valid box. (Note that we use the terms consistent and inconsistent to refer to the agreement between the valid box and the box winner, and not in reference to any non-stationary feedback state. The probabilities of the valid boxes were fixed throughout the task). Intuitively, in the consistent condition, participants had to learn that the player who won the valid box also won the entire round (i.e., by also winning one of the remaining two boxes), but in the inconsistent condition, participants had to learn that the player who lost the valid box won the entire round (i.e., by winning both of the remaining two boxes). Under a simple reinforcement learning account (e.g., Rescorla and Wagner, 1972), we should expect no difference between these conditions. For instance, Shanks (1987) found equivalent increases in judgments of causality in both positive and negative contingency (e.g., preventative causes) conditions (see also Danks et al., 2002). We further should expect no difference under a simple attentional learning account (since our manipulation is no different from an intradimensional shift; Kruschke, 1996).

By contrast, under a causal learning account, one might expect learning to be slower in the inconsistent condition than in the consistent condition. For instance, in the inconsistent condition, a player winning the valid box can be thought of as preventing that player from winning overall. Many studies have contrasted simple associative learning theories (usually instantiated as ΔP^1^), which typically only account for covariation of cues and outcomes, with more elaborate causal models (Cheng, 1997; Glymour, 2003; Griffiths and Tenenbaum, 2005). Many of these models predict equivalent learning between positive and negative contingencies. However, Danks et al. (2002) showed that slower learning in the negative contingency case is predicted whenever there is a prior bias toward expecting positive contingencies. Waldmann (2000, p. 54) phrases the problem thusly: are humans “sensitive...to causes and effects...or [can] they reduce learning events to cues and outcomes, which can give rise to mental representations that contradict physical reality”? In line with the assumption that participants learn via reasoning about causes, Waldmann (2000) demonstrated that when causes are presented after effects, people reason correctly about the causal direction contra simple associative learning models (see also Waldmann and Holyoak, 1992).

Having prior knowledge about the direction of the cue-criterion relationship also seems to allow people to form appropriate strategies and benefits learning (e.g., von Helverson et al., 2013). If participants in our tasks adopt a framing in which they link the winner of the box they’ve chosen with the overall winner, then this might lead to a bias in the expected causal relationship. Learning is faster and more efficient when expectations are consistent with the learning environment (Heit, 1994, 1997; Heit and Bott, 2000; Heit et al., 2004; Little et al., 2006). Consequently, learning in the inconsistent condition may have impaired relative to the consistent condition.

## Materials and Methods

### Participants

One-hundred and seven students from The University of Melbourne consented to participate in exchange for course credit. Participants were randomly assigned to each condition. There were 13 participants in each except in the 85% consistent and inconsistent conditions and in the 75% consistent condition where there were 14 participants. The experimental protocol was approved by the Human Ethics Advisory Group at the University of Melbourne.

### Procedure and Design

Participants were instructed that two computer-generated players were participating in a game that required each player to allocate (to the participant) an unknown number of tokens into three boxes. To win a round, a player had to have more tokens in at least two of the three boxes. On each trial, participants were allowed to reveal the contents of a box by selecting it with the computer mouse (**Figure 1**). Once selected, the number of tokens for each player was displayed, and participants then clicked on the player they thought won that round. Feedback (“correct” or “incorrect”) was immediately presented for 1500 ms. There were 10 practice trials, and 400 experimental trials divided into 10 blocks of 40 with a 750 ms blank interval between trials. Each participant completed the task alone in a sound-attenuated room.

Following precedent (Kruschke and Johansen, 1999; Little and Lewandowsky, 2009), participants were told that some boxes were more useful than others and that they should not always expect to be correct. Participants were also told that the answer could not be deduced mathematically. In reality, the number of tokens was randomly generated between 0 and 9 for each player; ties were not permitted in the valid box but could occur in the non-valid boxes. Participants were debriefed about the task afterward.

For each participant, only one box provided valid information. The valid box changed between participants. In addition, the frequency with which the valid box correctly predicted the winner varied between conditions from 75% to 100% of trials in four steps (i.e., 75, 85, 95, and 100%). Validity remained consistent throughout the experiment. For the non-valid boxes, the winning player was randomly selected with a probability of 0.5.

In the consistent (or inconsistent) condition, the player with the most (or fewest) tokens in the valid box was the ultimate winner with a frequency determined by the validity of the valid box.

## Results

**FIGURE 2:**
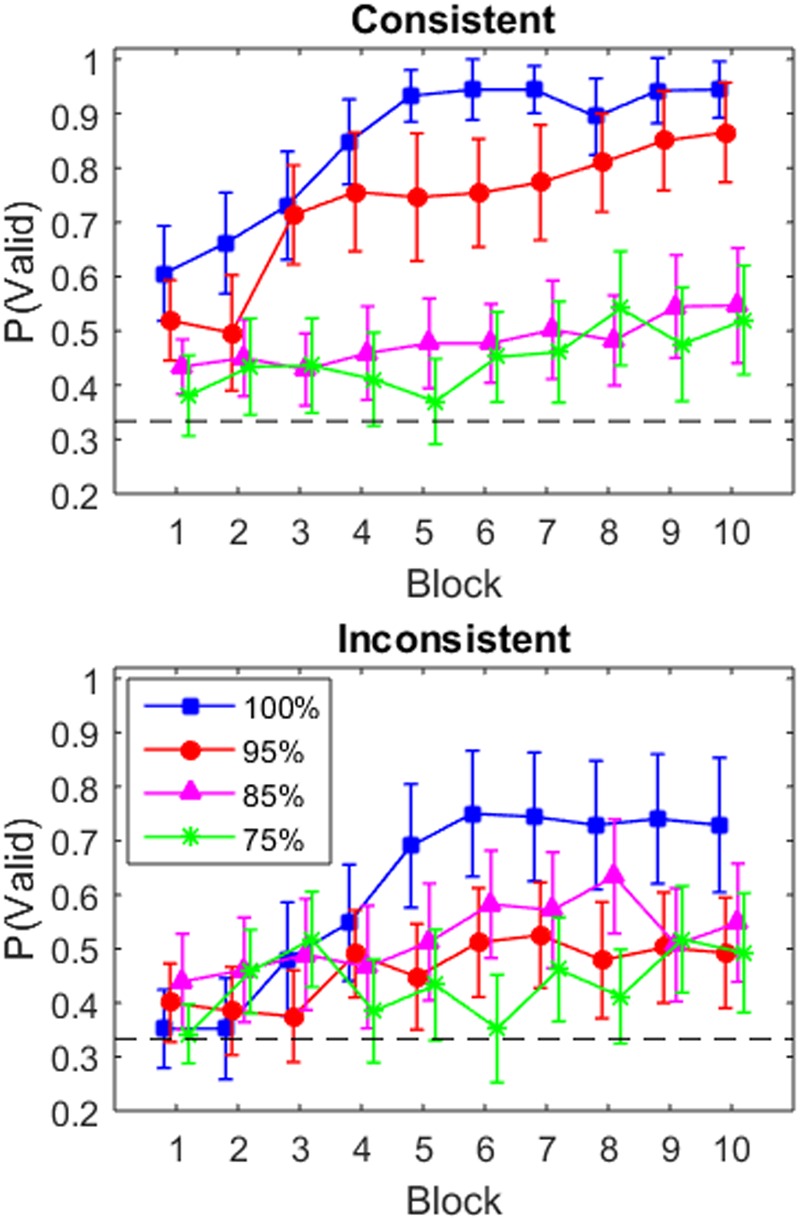
**Proportion of trials in which the participant selected the valid box, p(valid), in each block for the four validity conditions in the consistent condition **(Top)** and the inconsistent condition **(Bottom)**.** Error bars indicate one standard error. The dashed black line indicates chance performance in box selection (i.e., 1/3).

To determine whether participants learned to utilize the valid box, we created learning curves by averaging the proportion of valid box selection in each block (**Figure 2**).^2^ In the consistent condition (top panel), there is a clear separation between the 100 and 95% conditions, who show a rapid increase in utilization across block, and the 85 and 75% conditions, who show only a small increase (though performance is above the 33% chance level). In the inconsistent condition (bottom panel), the learning curves are more graded. In particular, the learning curves for the 100 and 95% conditions asymptote lower and are more variable than the same groups in the consistent condition. The 85 and 75% inconsistent conditions do not change substantially across blocks.

We transformed these proportions using an arcsine transformation to stabilize variances near ceiling by:

$$p\text{'}=2\cdot arcsine(\sqrt{p-[1/(2n)]})$$

where *n* is the number of observations on which the proportion is calculated (i.e., 40 observations per block per participant; Winer et al., 1971). Proportions less than 1/2*n* or greater than 1 - 1/2*n* were set to 1/2*n* or 1 - 1/2*n*, respectively, prior to the arcsine transformation. The resultant values were then subjected to a 10 Block × 4 Validity (100, 95, 85, or 75%) × 2 Consistency (consistent vs. inconsistent) between-within Bayesian ANOVA.^3^

As is evident in **Figure 2**, there is a main effect of block, a main effect of validity, and main effect of consistency. The Bayesian ANOVA (**Table 1**) confirmed this as Block, Validity, and Consistency each have BF_inc_ values greater than 1. However, the support for the consistency factor was weak (BF_inc_ = 1.22). There was also weak support for including the validity × consistency interaction (BF_inc_ = 1.33). We show in **Table 1** the models which, when their posterior probabilities are summed, account for 95% of the posterior probability. The three models always include the effects of block, validity, and the block × validity interaction. The model with the highest posterior probability also adds the main effect of consistency, and the model with the second highest posterior probability adds the validity × consistency effect.

**Table 1:** Results of a 10 Block × 4 Cue Validity × 2 Consistency Bayesian ANOVA on p(valid box) data.

	BF_inc_				
Effect	Overall	100%	95%	85%	75%
Block	>150	>150	>150	<1	<1
Validity	>150	–	–	–	–
Consistency	1.22	1.46	2.7	<1	<1
Block × Validity	>150	–	–	–	–
Block × Consistency	<1	<1	2.42	<1	<1
Validity × Consistency	1.33	–	–	–	–
Block × Validity × Consistency	<1	–	–	–	–

**Overall: Highest posterior probability General Linear Model (GLM)**					p(M| data)

Block + Validity + Consistency + Block × Validity					0.39
Block + Validity + Consistency + Block × Validity + Validity × Consistency					0.38
Block + Validity + Block × Validity					0.23

**100% Validity: Highest posterior probability GLM**					p(M| data)

Block + Consistency					0.67
Block					0.31

**95% Validity: Highest posterior probability GLM**					p(M| data)

Block + Consistency					0.43
Block + Consistency + Block × Consistency					0.38
Block					0.20

**85% Validity: Highest posterior probability GLM**					p(M| data)

Null model					0.64
Consistency					0.29
Block					0.05

**75% Validity: Highest posterior probability GLM**					p(M| data)

Null model					0.56
Consistency					0.25
Block					0.12
Block + Consistency					0.06

*Follow-up planned Block × Consistency Bayesian ANOVA results are also shown.*

*Models whose summed posterior probabilities account for at least 95% of the posterior probability are shown.*

To further characterize the results shown in **Figure 2**, we ran a series of 10 Block × 2 Consistency between-within Bayesian ANOVAs for each of the four validity conditions (**Table 1**). In the 100% condition, the main effects model (Block + Consistency) was the most preferred model (**Table 1**); the evidence for inclusion of the consistency variable is weak but positive, BF_10_ = 1.46. Likewise, in the 95% condition, the main effects (Block + Consistency) model was the most preferred model (in terms of having the highest posterior probability), but there was some evidence in favor of including the interaction, BF_inc_ = 2.42. In both the 85 and 75% validity conditions, the null model was preferred over all the alternative models. In both, the consistency-only model had the second highest posterior probability (**Table 1**).

Taken together, these results indicate that (a) probabilistic feedback substantially limits the ability to learn which box is the valid box, and (b) providing an inconsistent frame for understanding the task substantially decreases valid box selection. The latter finding may be due to a greater difficulty for participants to learn a negative contingency between box outcome and overall winner (cf. Betsch et al., 2016; see also Busemeyer et al., 1997; Rolison et al., 2011, for a description of similar results in the prediction of continuous criterion). A subsequent analysis was conducted to examine this possibility.

### Learning of Box Validity

To examine whether participants learned the contingency between the valid box and round winner, a second analysis focused on response selection in the second stage of the two-stage learning task. This analysis examined the likelihood that participants select the correct player choice (i.e., based on their condition) conditional on sampling from the valid or non-valid boxes. To avoid confusion, we term correct player choices as *rule-consistent* choices. Do participants in the consistent (or inconsistent) group learn to choose as the overall winner the player who had the most (or least) tokens in the inspected box? In general, participants who learned the overall consistent or inconsistent validity rule should make their player choice selection based on the appropriate outcome of the box selection – selecting the player with more tokens in the consistent condition and selecting the player with fewer tokens in the inconsistent condition.

**FIGURE 3:**
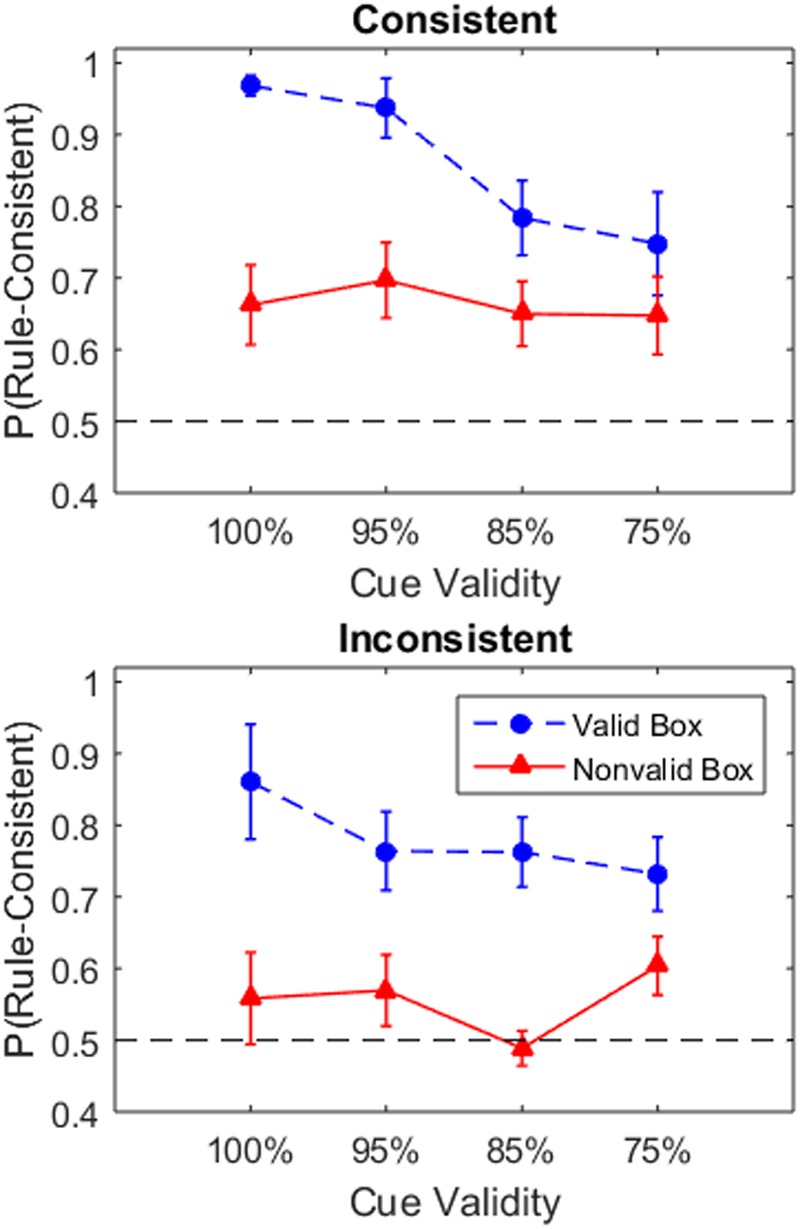
**Proportion of trials in which the participants selected the rule-consistent player after selecting from either the non-valid or valid boxes.** This indicates the frequency with which participants selected the player with most tokens in the consistent condition and player with fewest tokens in the inconsistent condition. Mean proportion was calculated for each participant and averaged across participants for each group. Error bars indicate one standard error. The dashed black line indicates change performance in player selection (i.e., 1/2).

**Figure 3** shows the mean proportion of rule-consistent choices after selecting the valid and non-valid box across conditions. Similar to the previous analysis, the data was analyzed using a three-way Bayesian ANOVA of 2 Box Choice × 4 Validity × 2 Consistency (**Table 2**). The three main effects and the Box Choice × Consistency interaction all had BF_inc_ values indicating moderate to strong support for their inclusion. The model which included these factors had the highest posterior probability, p(M_Box Choice + V alidity + Consistency + Box Choice × V alidity_ | data) = 0.53.^4^ This model was preferred over the models which also added the Validity × Consistency interaction, p(M_BoxChoice + V alidity+Consistency+BoxChoice×V alidity+V alidity×Consistency_—data) = 0.13, or the Box Choice × Consistency interaction, p(M_BoxChoice + V alidity + Consistency + Box Choice × V alidity + V alidity × Consistency_ — data) = 0.12. The next highest posterior probability was 0.06 for the model containing only the three main effects. All conditions learned to adopt the rule-consistent strategy when choosing the valid box. In the consistent conditions, participants also seem to adopt the rule-consistent choice at above chance level even when selecting the non-value box. By contrast, participants in the inconsistent condition were closer to chance after selecting the non-valid boxes reflecting the greater uncertainty about the correct strategy.

**Table 2:** Results of a 2 Box Choice × 4 Cue Validity × 2 Consistency Bayesian ANOVA.

Effect	BF_inc_
Box Choice	>150
Validity	5.27
Consistency	9.87
Box Choice × Validity	11.78
Box Choice × Consistency	<1
Validity × Consistency	<1
Box Choice × Validity × Consistency	<1

One caveat for this analysis is that the sampling rate of the valid and non-valid boxes was unequal between participants. Biased estimates of each participant’s propensity to select the rule-consistent player could arise due to having only encountered a small sample size of the valid and non-valid boxes. That is, a participant who exploited the valid box would have only a very small number of samples from the non-valid boxes. To test this possibility, we also computed the total proportions of rule-consistent player selections for each group by summing the frequencies across participants and dividing by the total sample for each group (**Table 3**). These results are generally in agreement with **Figure 3**.

**Table 3:** Mean proportion of selecting the rule-consistent player after selecting from the non-valid and valid boxes.

	Consistent				Inconsistent			
Box	100%	95%	85%	75%	100%	95%	85%	75%
Valid	0.97	0.96	0.84	0.85	0.96	0.84	0.84	0.78
Non-valid	0.67	0.60	0.60	0.61	0.58	0.58	0.50	0.56

*Rule-consistent refers to the player with the most tokens in the consistent condition and the player with the least tokens in the inconsistent condition.*

## Discussion

We found that participants in the inconsistent condition were less successful in utilizing the valid box than in the consistent condition especially at higher validity levels. This occurred despite the fact that the only variation between the two conditions was whether the likely overall winning player was the same player who had won or lost the valid box and that participants could achieve optimal performance by simply learning this contingency. Importantly, participants learned to differentiate between the valid and invalid boxes in both the consistent and inconsistent conditions. That is, participants were more likely to select the rule-consistent player after selecting the valid box. Therefore, poor utilization of the valid box in the inconsistent condition does not result from a failure to learn the negative contingency in that condition.

A possible explanation is that participants’ search behavior is driven by the opportunity to develop a coherent representation of the task (Gureckis and Love, 2009). In the consistent condition, the correlation between the winners of each box does not vary depending on which box is inspected allowing the formation of a coherent representation of these correlations. Conversely, in the inconsistent condition the correlations do vary depending on which box is inspected, thereby preventing the participant from creating a coherent representation.

To explain, to win the round, a player must win two boxes. In both conditions, the invalid box is not correlated with the winner of either of the remaining boxes. In the consistent condition, the participant learns that whichever player wins the valid box tends to win overall. Having won the valid box (e.g., having lower prices), the player only needs to win one of the two non-valid boxes (e.g., higher quality food but slower service). In the inconsistent condition, whoever wins the valid box tends to lose overall. Having won the valid box (e.g., lower prices), to lose overall the player would then need to lose both of the non-valid boxes (e.g., poorer quality food and slower service). To do so consistently implies that the invalid boxes must be correlated with each other (i.e., when you lose one you tend to lose the other) and negatively correlated with the valid box. In reality, the outcome of an invalid box is not correlated with the outcomes of either of the two other boxes or the overall outcome. Because the correlational relationship between the boxes varies depending on which box is inspected, the participant cannot develop a coherent representation in the inconsistent case. Or rather, if participants in the inconsistent condition adopted a causal model, they could never find consistent evidence for it as participants only saw the contents of one box per trial. Note that this was intended and not a flaw in the design.

Reducing cue validity impaired participants’ ability to exploit the valid box. Most interestingly, reducing task consistency produced the same effect. Dayan and Niv (2008) make a distinction between model-based learning, which emphasizes the construction of an internal model, which is used to support goal-directed action, and model-free reinforcement learning (i.e., Rescorla and Wagner, 1972; Sutton and Barto, 1998), which uses experience to estimate cue validities and payoffs without the construction of an internal model. These theories have been applied to similar two-stage choice tasks (Otto et al., 2013), and it is worthwhile to consider how they might apply as a *post hoc* explanation of our data. Model-free learning is not influenced by top-down hypotheses about the state of the environment, and involves areas associated with habitual action (Killcross and Coutureau, 2003; Balleine, 2005). Under this dichotomy, our task is one in which the model-free aspects of the task are identical between the consistent and inconsistent conditions, and thus no difference is expected under a reinforcement learning framework. However, the model-based representations of the contingencies between the tasks differ since participants derive different expectations or causal beliefs within each condition. Humans are clearly susceptible to interference with model-based representations though it is surprising that the decrement is so severe.

Our task is a natural complement to the task used by Avrahami and Kareev (2009). Avrahami and Kareev (2009) were concerned with how two competing opponents of unequal strength would distribute resources among various criteria when the assessment of those criteria was either deterministic or probabilistic. In their study, two players were given a number of tokens to distribute amongst a number of boxes. The total number of tokens was uneven such that one player was “stronger” than the other. In this classic variant of the Colonel Blotto game, the winner of any round was determined by selecting one of the boxes and determining which player had placed more tokens in that box. It is clear that if a ‘referee’ repeatedly and deterministically checks one of the boxes, then the stronger player will learn over time which box is consistently assessed, so will distribute all their tokens to this box and consequently always win the game. By contrast, if the referee selects boxes probabilistically then the weaker of the two players can occasionally win the game by foregoing some of the boxes and allocating more of their meager allotment to fewer boxes. This is, in fact, the optimal solution to this task, and participants approximated this solution well under probabilistic conditions.

In our task, the tokens were generated with a fixed probability structure. In Avrahami and Kareev’s task, the computer referee chose a box with a fixed probability structure. It is therefore interesting to consider generalizations of both designs in which there are two human players and one human referee. It is likely that a number of possible states would evolve out of the three-way interaction between the players with each player utilizing dynamic rather than static strategies. Two likely possibilities are oscillatory states that cycle through different configurations of allocation and evaluation strategies and steady states that settle into deterministic allocation and evaluation. The emergence of these states would likely depend on relative strength of the players and individual differences such as working memory capacity, which influences learning rate and strategy use (Lewandowsky, 2011; Sewell and Lewandowsky, 2012). Future research should determine the nature of how these individual differences interact with the cue structure to lead to different patterns of performance.

In decision-making studies, it is common to incentivize participants with monetary rewards in order to motivate high performance and profit maximization. Participants in the current study, however, only received course credit for their performance and this may limit the generalizability of the current findings. Nevertheless, participants were sufficiently motivated to demonstrate increased utilization of the valid box across blocks and discrimination the valid from the non-valid boxes. Critically, our findings illustrate that participants failed to utilize more valid information within an inconsistent task structure. The lack of monetary reward, however, may encourage participants to explore non-valid boxes since there is no financial cost for this behavior, and thus this issue should be explored in future studies. A second limitation of the present task is that although participants were informed of the probabilistic nature of the task upfront, participants in the inconsistent condition were not informed about the difficulty of forming a causal model of the task. By our walking on the slippery slope between deception and not providing complete information about cue generation, we may have inadvertently increased participant frustration, which may have altered behavior. Nonetheless, we feel that the current study offers insight to how task structure and participant expectation can affect learning and exploratory behavior within a probabilistic learning environment.

## Author Contributions

PH, DL, and NC planned and designed the study, with NC completing the data collection. TW and DL completed the majority of the data analyses and first draft of the manuscript. All authors contributed to the writing.

## Conflict of Interest Statement

The authors declare that the research was conducted in the absence of any commercial or financial relationships that could be construed as a potential conflict of interest.

## Footnotes

1 ΔP is calculated as p(outcome | cause present)-p(outcome | cause absent) and can be thought of as the asymptotic weight of a causal stimulus trained using a simple associative learning model (e.g., the delta rule; Rescorla and Wagner, 1972).

2 We report an analysis of individual differences in the Supplementary Material.

3 All ANOVA results were computed using JASP (Rouder et al., 2012; Love et al., 2015; Morey and Rouder, 2015) These tests utilize Bayes factors (BF) for comparing General Linear Models (GLMs) that either include or omit each effect or interaction. These analyses use the generalized Cauchy priors over standardized effects to form “default” BF tests (see Rouder et al., 2012, for details). The null hypothesis is implemented by assuming that the coefficient on a particular GLM factor equals zero. The BF_10_ then provides the ratio of the null hypothesis model’s evidence compared to the alternative hypothesis which assumes that the coefficient for a particular component does not equal 0. Hence, the BF_inc_ for the inclusion of a factor can be interpreted in the same manner as a traditional Null Hypothesis test (but can be interpreted as evidence either for or against the null hypothesis). We follow Kass and Raftery’s (1995) interpretation such that any BF less than 1 indicates support for the null hypothesis, and 1 < BF < 3, 3 < BF < 20, and 20 < BF indicate weak, strong, and very strong support for the alternative hypothesis (Jeffreys, 1998).

4 Technically, the posterior probability is also conditional on the model that we adopted for the likelihood and the prior probability distributions, outlined in Footnote 3, over the model parameters in the likelihood. We omit the conditionalization on the model for simplicity.
