# Supplementary material for: Global Cue Inconsistency Diminishes Learning of Cue Validity
Source: Front Psychol. 2016 Nov 11;7:1743. doi: 10.3389/fpsyg.2016.01743 (PMC5104735; doi:10.3389/fpsyg.2016.01743)
Supplement: Supplementary file 1 [file Data_Sheet_1.pdf]

Individual Differences Clustering Analysis

To analyse the individual differences in each condition, we used a two stage clustering process by first applying a hierarchical Bayesian quantitative clustering algorithm and then second by extracting theoretically interesting clusters which captured qualitatively different performance. For the first step, we applied the clustering analysis method introduced by Navarro, Griffiths, Steyvers, and Lee (2006) for identifying latent groups of participants who exhibit qualitatively different response strategies. One benefit of this approach is that it allows for maximizing within-cluster similarities and minimizing between-cluster similarities. This means that that true performance is not obscured by averaging over qualitatively different response strategies, but the potential "signal-boosting" benefits of averaging over qualitatively similar response strategies are retained. A second benefit of the approach is that it allows for a potentially infinite number of latent groups; hence, the number of groups need not be specified in advance. In a nutshell, the method works by setting Dirichlet process prior over the mixing weights for the likelihoods for each group; the likelihood for each group is determined by the specific statistics of that group. In our application, we clustered participants in each condition on the frequency with which they chose the valid box across the ten experimental blocks.

More specifically, the basic assumptions underlying the use of a Dirichlet process prior over the weights in a mixture model are that a) the number of latent groups is unknown but can be estimated from the data if we assume that b) the probability of the joining a group is proportional to the size of the group and that c) the probability of forming a new group is proportional to a parameter, α. The posterior distribution over the group assignments, g_i (i.e., the group index of data point *i*) can be estimated using Gibbs sampling with the following conditional probabilities (see Navarro et al., 2006):

$$p\left(g_i = G \mid g_{-i}, \alpha, x\right) \propto p\left(g_i = G \mid g_{-i}, \alpha\right) p\left(y_i \mid g_i = G, g_{-i}, y_{=i}\right) \quad (1)$$

The first term on the right hand side is a Dirichlet Process prior given by:

$$p\left(g_i = G \mid g_{-i}, \alpha\right) \propto \begin{cases} \dfrac{n_G}{n-1-\alpha} & \text{if } n_G > 0, \\ \dfrac{\alpha}{n-1-\alpha} & \text{otherwise.} \end{cases} \quad (2)$$

The second term, the predictive likelihood of data point *i* given the other data points from group is the posterior probability of observing parameter value y_i given the parameters already observed in group *G*. We assume that the predictive likelihood can be found by integrating over the parameters of the distribution of the group members, which we assume to be distributed as a multinomial.

The clustering analysis revealed 20 clusters across all 2 consistency x 4 validity conditions. The clusters were not uniformly distributed across conditions with more clusters being identified in the inconsistent condition and the lower validity conditions. 81% of subjects were assigned to five groups, the remaining clusters tended to contain only one subject whose performance was highly idiosyncratic. It is worth noting that these singleton clusters tended to appear in the lower validity conditions.

Inspection of the clusters revealed that many of the clusters captured similar qualitative patterns of performance. These patterns of performance could be generally classified into six types: (1) better than chance performance with little change over the course of the blocks (N = 60), (2) fast learning with a high asymptote (N = 15), (3) slower learning with a high asymptote (N = 12), (4) slow learning with a lower asymptote (N = 4), (5) reverse learning or poorer than chance performance (N = 10), and (6) idiosyncratic performance (N = 6). In the first three clusters, participants learned the valid box to a high level of performance (see Figure S1). The remaining clusters are shown in Figure S2.

Summary

The clustering analyses reveal the emergence of a number of different strategies with the emergences of some strategies more prevalent in some conditions than others. These different strategies reflect a number of different facets of performance including fast learning of the appropriate cues, slower incremental learning, and above chance stable performance with little change in accuracy across blocks. Conditions with coherent tasks representations and higher validity were better able to develop a coherent model of the task and exhibit higher levels of performance. Participants in the remaining conditions were able to learn the task better than chance but were impaired either due to decreased cue validity or the inconsistent nature of the task.

References

Navarro, D. J., Griffiths, T. L., Steyvers, M., & Lee, M. D. (2006). Modeling individual differences using Dirichlet processes. *Journal of Mathematical Psychology*, *50*(2), 101-122.

Figure Captions

Figure S1. Proportion of trials in which participant selected the valid box, p(valid), in each block for several high performing clusters identified by our clustering analysis. The top panel shows Cluster 1, which we identified as containing subjects who have better than chance performance with little change over the course of the blocks; the middle panel shows Cluster 2, which we identified as containing participants who showed fast learning with a high asymptote; and the bottom panel shows Cluster 3, which we identified as containing participants who showed slower learning but to a high asymptote. Each line shows the group of participants from each consistence condition (C = consistent, I = inconsistent) and validity condition (100, 95, 85 & 75%). Participant counts are given by N.

Figure S2. Proportion of trials in which participant selected the valid box, p(valid), in each block for several idiosyncratic clusters identified by our clustering analysis. The left panel shows Cluster 4, which we identified as containing subjects who have slow learning with a low asymptote; the middle panel shows Cluster 5, which we identified as containing participants who showed reverse learning or worse than chance performance; and the right panel shows Cluster 3, which we identified as containing participants who showed idiosyncratic (or unclassifiable) performance. Each line shows the group of participants from each consistence condition (C = consistent, I = inconsistent) and validity condition (100, 95, 85 & 75%). Participant counts are given by N.

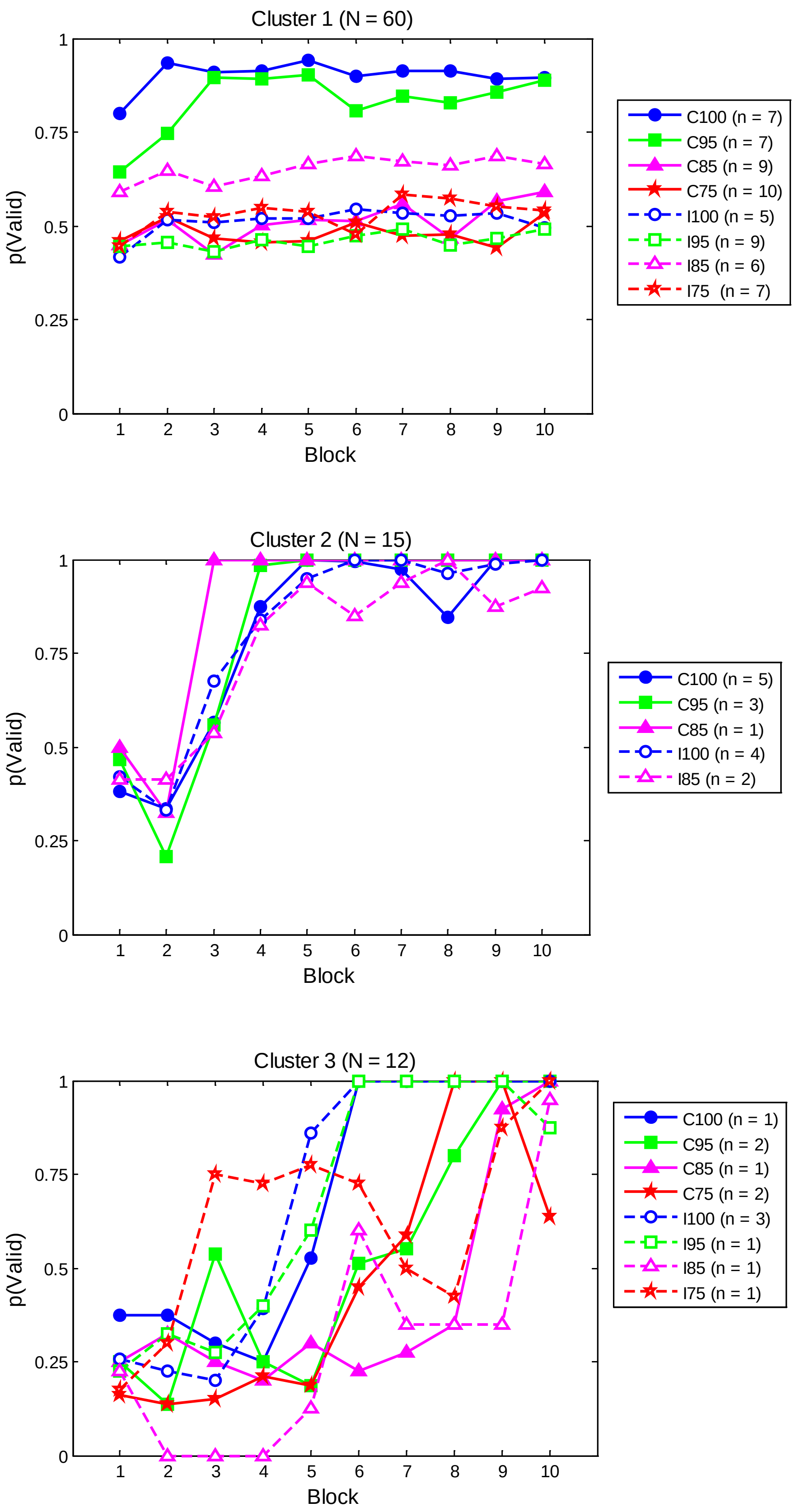
Cluster 1 (N = 60)
p(Valid)
Block
C100 (n = 7)
C95 (n = 7)
C85 (n = 9)
C75 (n = 10)
I100 (n = 5)
I95 (n = 9)
I85 (n = 6)
I75 (n = 7)
Cluster 2 (N = 15)
p(Valid)
Block
C100 (n = 5)
C95 (n = 3)
C85 (n = 1)
I100 (n = 4)
I85 (n = 2)
Cluster 3 (N = 12)
p(Valid)
Block
C100 (n = 1)
C95 (n = 2)
C85 (n = 1)
C75 (n = 2)
I100 (n = 3)
I95 (n = 1)
I85 (n = 1)
I75 (n = 1)

Figure S1

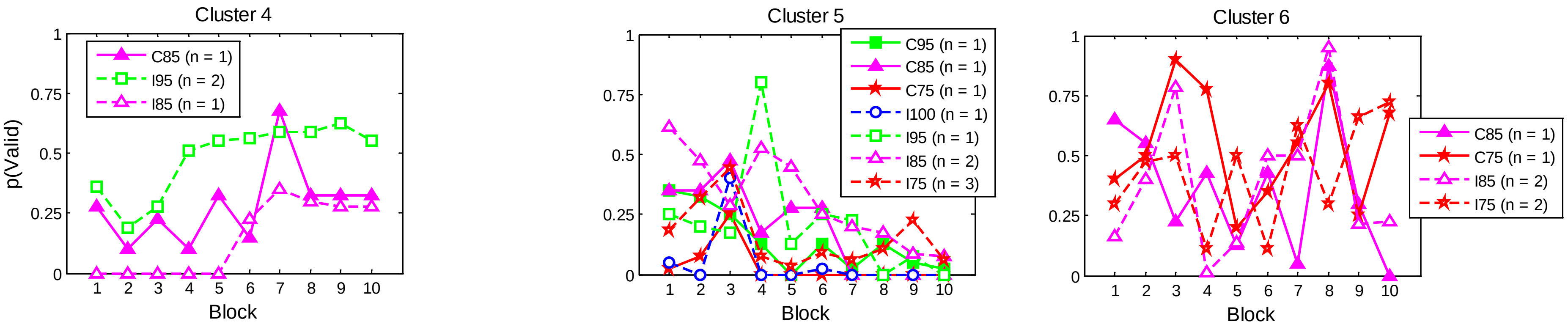
Cluster 4
Cluster 5
Cluster 6
p(Valid)
Block
C85 (n = 1)
I95 (n = 2)
I85 (n = 1)
C95 (n = 1)
C85 (n = 1)
C75 (n = 1)
I100 (n = 1)
I95 (n = 1)
I85 (n = 2)
I75 (n = 3)
C85 (n = 1)
C75 (n = 1)
I85 (n = 2)
I75 (n = 2)

Figure S2
